# Comparative Evaluation of Two Obstetrical/Gynecology Resident “Boot Camps” of Different Lengths: Equivalent Practice Skills Confidence and Knowledge Levels

**DOI:** 10.51894/001c.7114

**Published:** 2019-03-04

**Authors:** Jeffrey D. Postlewaite, David Boes, Salvatore Finazzo, Cammie Cantrell, William D. Corser

**Affiliations:** 1 Metro Health-UM Health Hospital, Wyoming, MI; 2 Henry Ford Wyandotte Hospital, Wyandotte, MI; 3 Executive Assistant, Health Management Associates, Lansing, MI; 4 MSU Statewide Campus System, College of Osteopathic Medicine. East Lansing, MI

**Keywords:** knowledge improvements, practice confidence, ob/gyn, resident boot camps

## Abstract

**CONTEXT:**

Since the earlier time of master-apprentice type GME relationships, more residency program educators have developed various forms of *boot camps* to ease incoming learners into their new specialty roles as first-year residents. Such boot camps have ranged from informal informational sessions with faculty using simulation activities, to more formal workshops entailing pre- and post-event skills assessments with simulation exercises, formative feedback and debriefing sessions. The purpose of this pilot project was to examine for relative pre- and post-boot camp changes in Obstetrics/Gynecology (OB/GYN) practice skills confidence and knowledge levels in two consecutive cohorts (2014 and 2015) of first-year residents.

**METHODS:**

Boot camps were of two different lengths: a five-day 2014 camp (n = 32 residents) and shortened three-day 2015 boot camp (n = 29 residents). Respondents from both boot camp cohorts were invited to complete the same 25-item OB/GYN practice skills confidence and knowledge survey. The first three authors developed this survey prior to the initial boot camp (2014). Revisions/adjustments were then made to content after the 2014 to pare down from the initial five days’ worth of content for the 2014 boot camp to three days for the 2015 boot camp.

**RESULTS:**

Each of 45 sample resident respondents who provided complete pre-and post-boot camp data demonstrated improvements in self-rated practice confidence and knowledge levels. Mean per resident pre-post-boot camp survey rating levels for individual items in the shorter 2015 boot camp cohort increased by 1.096 (SD = 0.5487), over a two-fold increase for most individual items in the 2014 residents. Mean cohort differences represented a non¬-significant equivalent increase in pre-post practice confidence and knowledge levels for individual ratings items between the 2014 and 2015 cohorts (p = 0.241).

**CONCLUSIONS:**

Based on these preliminary results, the authors conclude that it may be possible to adjust their OB/GYN boot camp from five days to three and still achieve comparable learner outcomes while delivering the same basic content.

## INTRODUCTION

Throughout their undergraduate and postgraduate medical education as learners, most incoming resident physicians face a series of transitions that can be stressful, sometimes causing them to question the adequacy of their clinical skills as they enter graduate medical education (GME) training. The major transition entailed from completing a medical school program to starting a specialty residency program may cause many new residents to experience uncertainty or anxiety concerning their preparation to enter clinical practice.^1–3^

Since the earlier time of predominant master-apprentice type GME relationships, more residency program educators have developed various forms of *boot camps* to ease incoming resident learners into their new/prospective specialty roles as first-year residents.^1,3–12^ As has been noted in one meta-analysis, one proposed definition is “A boot camp is a focused course designed to enhance learning, orientation, and preparation for learners entering a new clinical role.”^11^

To date, boot camps have ranged from informal informational sessions with faculty, using some types of simulation activities, to more formal workshops entailing pre and post-event needs and skill assessments with simulation exercises, formative feedback and debriefing sessions.^10–13^ Although the development and delivery of simulation-based boot camp sessions has increased, there continues to be a relative shortage of published studies which have systematically evaluated how various boot camp formats might be associated with GME learner outcomes.^4,6,8,10,11^

For over 25 years, the Statewide Campus System^14^ (SCS) in the Michigan State University College of Osteopathic Medicine has coordinated the educational offerings for resident physicians and faculty across the state. The SCS currently serves over 190 community-based residency programs in 37 affiliated healthcare systems. In 2014, the SCS-affiliated clinician authors of this paper (JDP, DB, SF) initiated a five-day Obstetrics & Gynecology (OB/GYN) skills and competency boot camp in an effort to better prepare a sample of 32 first-year resident learners from 13 osteopathic-oriented Michigan programs.

Although the feedback from the first 2014 boot camp participants was positive, the OB/GYN authors (JDP, DB, SF) felt that it may be prudent to shorten the 2015 boot camp based on the logistical (e.g., resident and faculty schedules, venue costs, etc.) complexities of delivering the longer event for learners from across the state. After reviewing the boot camp content, it was determined they could cover the same content in three days specifically by minimizing content duplication.

### Purpose of Analyses

The purpose of these exploratory descriptive analyses was to examine for relative pre and post-boot camp changes in OB/GYN practice skills confidence and knowledge ratings in two cohorts of first-year OB/GYN residents post completion of boot camps of two different lengths: either a five-day boot camp (July, 2014) or a shortened three-day boot camp (July, 2015).

## METHODS

The first 2014 boot camp was established by the first four authors (JDP, DB, SF, CC) to run for five consecutive days for first-year incoming residents from one of 13 OB/GYN Osteopathic Residencies in Michigan. The number of first-year residents in each residency program ranged between two and four, and the SCS provided a mechanism (website calendar downloadable pdf’s) to share OB/GYN pre-boot camp educational resources.

The 2014 boot camp was structured to encompass an introduction to technical OB/GYN practice skills and knowledge content to help residents develop confidence in the transition from medical school to residency. Because our incoming resident learners come from around the country, it also allowed the residents to self-assess how confident they felt in their OB/GYN practice skills and knowledge compared to their colleagues from different medical school backgrounds.

This 2014 five-day boot camp encompassed didactic presentations, various knot tying skills, episiotomy/perineal laceration repair, a variety of obstetrical skills (with a full day in the campus-based simulation center), OB/GYN triage cases, and quality and safety in the hospital. There was intentional repetition with some of the planned skills training to encourage retention. Fetal heart rate (FHR) interpretation was also a component of this boot camp. In 2015, the authors had decided to consolidate most of the same basic content into a three-day boot camp. Participation in both boot camps was encouraged but not mandatory.

Respondents from both the 2014 and 2015 boot camp cohorts were invited to complete the same 25-item OB/GYN practice skills confidence and knowledge survey that had been developed by the first three authors (JDP, DB, SF) before the first 2014 boot camp. (Appendix 1) The survey items each used a 1-5 Likert-type scale ranging from “Strongly Disagree” to “Strongly Agree” with an open-ended “comments” item at the end of the survey for respondents to enter any comments and/or suggestions for future boot camps.

The de-identified pre- and post-boot camp survey data from both OB/GYN cohorts were entered by the analyst author (WDC) into an S.P.S.S. version 22^15^ data set for comparative descriptive analyses.

## RESULTS

Complete pre- and post-boot camp OB/GYN practice skills confidence and knowledge ratings data were obtained from a total of 45 resident respondents, 33 from the 2014 boot camp and an additional 12 respondents from the 2015 boot camp. Quantitative survey item data required only a minor amount of cleaning. A total of 28 open-ended qualitative comments or suggestions written in by residents were also entered into word processing software.

### Mean Pre-Post Boot Camp Practice Skills Confidence and Knowledge Differences

Each of the total 45 resident respondents demonstrated an overall improvement in pre- to post-boot camp practice skills confidence and knowledge levels. Mean per resident pre-post boot camp responses for the shorter 2015 boot camp increased by 1.096 (SD = 0.5487) on the 1 through 5 scale, indicating that most members of the 2015 resident cohort respondents rated themselves on average as “more confident” in the specific OB/GYN skill and knowledge areas for most items than 2014 residents demonstrating a mean 0.0453 (SD = 0.1628) survey item increase. When comparing the two cohorts, mean “between group” differences were not found to be statistically significant (i.e., overall equivalent) (p = 0.241).

Figure 1 depicts the overall equivalent distributional patterns of practice skill confidence and knowledge improvements measured from the two learner cohorts. The larger number of red-colored 2014 respondents exhibit a similar distributional pattern of score improvements when compared to the smaller number of light blue-colored 2015 residents. Since they were using a newly created survey instrument, the authors avoided trying to calculate any type of composite survey score until the survey had undergone some additional refinement and possible psychometric testing in future studies.

**Figure:**
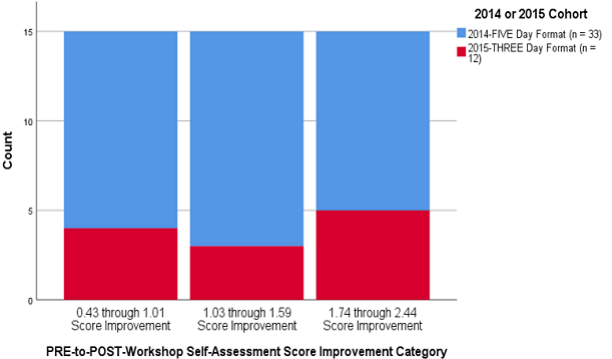
Figure 1 Comparison of 2014 to 2015 Pre-Post Boot Camp Practice Skills Confidence and Knowledge Item Improvements

### Individual Practice Skills Confidence and Knowledge Item Improvements

As might be expected, the average individual resident practice skills confidence and knowledge item score increased for every item in pre and post-boot camp surveys for both cohorts. However, the following six items were those that increased the most when comparing the 2014 to 2015 boot camps:

1. *“I can discuss the fire risk score and what it means to us and our patients;”* (mean increase from 2.430 (in 2014) to 2.600 (in 2015)
2. *“I am able to competently repair simulated lacerations of the perineum and simulated episiotomies;”* (*mean* increase from 1.980 to 2.370)
3. *“I understand the Duty Hour Rules, how to log them, and their significance;”* (*mean* increase from 2.200 to 2.400)
4. *“I can describe and perform the closure of first degree midline episiotomy;”* (mean increase from 1.890 to 2.160)
5. *“I can describe the important components of”handoffs” and their importance;“* (*mean increase from 1.850 to 2.030*)
6. *“I am aware of the Statewide Campus System Research Modules.”* (*mean increase from 2.170 to 2.440*)

### Qualitative Resident Comments

A total of 28 (62.2% of complete data sample) respondents offered specific comments regarding what components of their respective boot camp they most appreciated or could be improved.

### 2014 Five-Day Boot Camp

### Strengths

- Great hands on learning opportunities.
- Suturing practice every day was helpful.
- Made me less apprehensive going into intern year.
- Fetal monitoring course was awesome.
- Great review of instruments.
- Laid back lectures helped information get across.

### Areas to improve

- Need more instruction on fetal heart rate monitoring steps.
- A few very repetitive lectures (Labor & Delivery).
- NEED to go over suturing in a lecture before going into hands on.
- Could be condensed to 3-4 days without all of the repetitive lectures.
- More time for Neonatal Resusciation Program (NRP).

### 2015 Three-Day Boot Camp

### Strengths

- I did enjoy the lecture that preceded today’s lab verses yesterday because it had pictures to help us visualize.
- Excellent amount of information. I really feel it was an immense help and a great way to begin breaking into residency.
- Triage cases were the most helpful today.
- The clinical based lectures and hands on experiences we did have were very beneficial.

### Areas to Improve

- I would have liked to have heard the lecture about suture types and uses of each. I feel like I am very weak in that area. I also would have liked the amniotomy drill.
- An overview of the different types of sutures and what is commonly used in different parts of C-sections, etc. would have been very helpful.
- Vaginitis lecture was good but was primarily a review.
- I would have liked for these three days to revolve around clinical education and simulated skills labs.
- In general more hands on and less lecture.
- In regard to future boot camps - a formal ultrasound lecture with images and ideally videos would be very helpful. Also, it would have been great to have the lab for amniotomy, (intrauterine pressure catheter) (IUPC), and fetal scalp electrode (FSE).

## DISCUSSION

During recent years, a wide variety of sizes, types, and duration of boot camps have evolved for different types of first-year physician residents.^1,3,6,10^ The boot camps to date have lasted from one day to seven weeks, with a wide variation in number of hours per day, and/or days per week. Nevertheless, most reports suggest these comprise an effective tool to improve practice skills confidence and knowledge, with positive feedback from learners.^11^ The authors’ intentions for these boot camps were to develop an effective tool for transition from medical school to an OB/GYN residency. This was considered particularly important given the typical time constraints between graduation and starting residency as earlier described.

The overall goal of these analyses was to determine if the authors could cover the same basic content in three days and achieve equivalent learner practice skills confidence outcomes. In future boot camps, we intend to utilize additional assessment tools (pre- and post-boot camp), such as the Association of Professors of Gynecology and Obstetrics (APGO) *Preparation for Ob-Gyn Residency Knowledge Assessment Tool* (PrepForRes) exam,^16^ to more rigorously measure effectiveness of learning and retention.

### Study Limitations

These results should be reviewed within the context of several major limitations. Obviously, two small convenience samples of OB/GYN residents from Michigan residency programs may certainly limit the generalizability of these findings to other parts of the country. In addition, the considerably smaller size of the 2015 respondent cohort limited our ability to use inferential statistical procedures to compare learner practice skills confidence and knowledge differences between the two boot camp cohorts.

This was also the first two times that the authors had used this untested 25-item survey instrument. The manner in which the 2014 and 2015 boot camps may have differed in other unmeasured ways could also have skewed our measured cohort differences. It would also have been ideal to follow these participating OB/GYN residents longitudinally to more systematically evaluate the actual total impact of the boot camp through the perspectives of the residents and/or their residency program faculty.

## CONCLUSIONS

There appears to be a growing consensus that there is benefit to providing a boot camp format for transition from medical school to residency.^5,6,11^ In addition, the Level 1 ACGME Milestones now provide a more focused set of expectations of what an incoming first-year OB/GYN resident should know or be able to perform.^17^ As various boot camp formats evolve, it will be important to measure the perceived value of these events by varied GME learners in addition to measuring pre- and post-training practice skill confidence levels.

Based on these initial results, the authors have determined that it may be possible to adjust the boot camp described here from five days to three and still achieve comparable practice skill confidence and knowledge outcomes while delivering the same basic OB/GYN content. While there has been a perceived benefit of providing a longer, more extensive boot camp, GME educators’ ability to provide longer multi-day events may be increasingly limited particularly by resource constraints.^11,12^

Additional studies with larger resident samples of OB/GYN residents and faculty are needed to examine the most cost-effective formats and lengths of boot camps currently offered to first-year residents across the nation. Ideally, the results of these small pilot analyses can help inform the future development and testing of boot camp events to facilitate new OB/GYN residents practice preparation.

The review of this manuscript was coordinated by SMRJ Assistant Editor Sam Wisniewski.

### Conflict of Interest

The authors declare no conflict of interest.

**Table:** Appendix 1 OB/GYN Boot Camp Resident Practice Skills Confidence/Knowledge Items*

1. I am aware of the Statewide Campus System Research modules
2. I am well versed on the expectations of social media as it pertains to professionalism in medicine.
3. I can discuss the components involved in the conduction of normal labor and delivery.
4. I am well versed in the instrumentation utilized in vaginal deliveries.
5. I am well versed in the names and function of the instruments used in a cesarean birth.
6. I am able to identify the different types of suture materials.
7. I can discuss the different uses of the different types of suture materials.
8. I can perform well-done one- and two-handed square knot ties.
9. I am able to competently repair simulated lacerations of the perineum and simulated episiotomies.
10. I am able to competently perform interrupted, figure of eight, running and running interlocking wound closures.
11. I am able to describe the steps in preparation for surgery and appropriate hand washing, gloving and gowning techniques.
12. I can describe three aspects of an institution that utilizes high reliability standards and its beneficial effects on patients and staff.
13. I can discuss the fire risk score and what it means to us and our patients.
14. I can describe the components of “OR Time out” and how this affects patient’s safety.
15. I can discuss the importance and components of e-Logs.
16. I understand the Duty Hour Rules, how to log them, and their significance.
17. I can describe the important components of “handoffs” and their importance.
18. I can describe the physiology of EFM and the current nomenclature to describe Electronic Fetal Monitoring tracings.
19. I can describe the pathophysiology of abnormal Electronic Fetal Monitoring tracings and the appropriate physician response to those abnormalities.
20. I can describe and perform the closure of 1^st^ degree midline episiotomy.
21. I can describe and perform the sequence of events for fetal scalp electrode placement, intrauterine pressure catheter placement, and amniotomy.
22. I can describe the appropriate components of cervical exam and appropriate documentation.
23. I can discuss how to document rupture of membranes.
24. I can discuss proper microscope usage and how to diagnose and treat common vaginitis encountered in OB/GYN.
25. I can describe the techniques involved in evaluation of the obstetrical patient that presents to triage for evaluation.

* 1 to 5 Likert-type scale ranging from 1 = “Strongly Disagree” to 5 = “Strongly Agree”
